# Coalescence and Break-Up Behaviors of Nanodroplets under AC Electric Field

**DOI:** 10.3390/molecules28073064

**Published:** 2023-03-29

**Authors:** Fenhong Song, Ruifeng Chen, Gang Wang, Jing Fan, Hu Niu

**Affiliations:** School of Energy and Power Engineering, Northeast Electric Power University, Jilin 132012, China; fenhongsong@neepu.edu.cn (F.S.);

**Keywords:** electro-coalescence, separation, molecular dynamic simulation, electric field

## Abstract

Water must be separated from water-in-oil (W/O) emulsion because of the corrosion it brings to the relative equipment in the process of transportation and storage. It is an effective method to apply external electric field to achieve high performance of separating small, dispersed water droplets from W/O emulsion; however, the coalescing micromechanism of such small salty droplets under AC electric field is unclear. In this paper, molecular dynamics simulation was adopted to investigate the coalescence and separation process of two NaCl-aqueous droplets under AC electric field and discuss the effect of AC electric field frequency, as well as the time required for contacting, the critical electric field strength, the dynamic coalescence process and the stability of the final merged droplet. The results show that the critical electric field strength of the droplet coalescence increases with the increase of frequency, while the time required for droplet contacting becomes shorter. The shrinkage function curve was applied to characterize the droplet coalescence effect and it was found that the droplets coalescence and form a nearly spherical droplet under the AC electric field with a frequency of 1.25 GHz and strength of 0.5 V/nm. When the electric field frequency is 10 GHZ, the merged droplet presents a periodic fluctuation with the same period as the AC electric field, which mainly depends on the periodic movement of cations and anions under the AC electric field. The results can provide theoretical basis for the practical application of electrostatic demulsification technology in the petroleum or chemical industry from the microscopic perspective.

## 1. Introduction

At present, as many oil fields in the world start to enter a high moisture content period, the water content of the crude oil produced increases gradually. In the process of transportation, storage and reprocessing after exploitation, the relative equipment might be corroded, resulting in the reduction of the working efficiency and the service life of the equipment. Therefore, water in the W/O emulsion must be removed by some techniques for crude oil dewatering, such as swirling method [1], ultrasonic method [2], heating sedimentation [3] and electrostatic coalescence etc. Among them, electrostatic coalescence refers to the technology subjected to the application of an electric field to the W/O emulsion, such as the DC electric field [4,5,6], the AC electric field [7,8] and the high-voltage pulse electric field [9], the small water droplets in the emulsion could move, deform and coalesce under the effect of the electric fields, resulting in a significant higher dewatering efficiency than the other conventional approach.

Much research was conducted to reveal the required conditions to coalesce and effectively improve dewatering efficiency [10,11,12,13,14,15]. An electric field with a strength smaller than the critical value could impact the coalescence and could increase the coalescing speed of the droplets. Meanwhile, the electric field above the threshold value could cause the instability of droplets and result in some partial break-up phenomenon or complete noncoalescence. Similarly to the external electric field, another impacting factor, the contacting cone angle also have been found to have a critical value [16]. The two contacting droplets coalesce completely if the contacting cone angle is smaller than the critical one, and they will otherwise separate directly or separate after partial coalescence in the case with a contacting cone angle larger than the threshold value. Additionally, Hamlin et al. [17] reported the existence of a critical ionic conductivity below which the oppositely charged drops only partially coalesced. Some other impact factors playing important roles in the coalescence process were also investigated in recent years. Guo et al. [18,19] studied the coalescence behavior of two water droplets under DC and AC electric field experimentally using a high-speed imaging. The results indicated that the droplets were easier to coalescence with smaller radius or under lower electric field strength and the individual droplet has a minimal deformation under AC electric field E = 300 kV/m with a frequency of 50 Hz. Abbasi et al. [20] investigated the effects of electric capillary number, droplet size, and the distance between droplets on dynamic droplet pair coalescence and its stability under a DC electric field. These experiments and numerical simulations investigated the electrical coalescence from a macroscopic perspective. When the size of the water droplet in W/O emulsion comes to several nanometers, it is difficult to explore the micro-mechanism of coalescence in such a small scale through the traditional macroscopic method.

Molecular dynamics method describes the interactions between atoms in the simulation system based on some potential functions with the accuracy parameters, and provides a powerful tool to study nano/micro phenomena from a molecular level [21,22,23,24,25]. It can obtain the location information and capture any instantaneous state of droplets during the coalescence process and the forming process of the liquid bridge. It can be adopted to investigate the deformation, migration, coalescence and breaking-up characteristics of salty droplets from a microscopic perspective. Li et al. [26,27] systematically discussed influences of electric field strength, droplet diameter, nanoparticle (NP) concentration, NP size and electric frequency on the nanoparticle-laden droplet–droplet coalescence behaviors under DC/AC electric fields, and also found the existence of the critical contacting cone angle. Chen et al. [28] investigated the effect of a DC electric field on the coalescence and breakup behaviors of binary emulsion nanodroplets and found that the efficiency of the electrocoalescence was promoted by raising the electric field strength. Another simulation result reported by Li et al. [29] showed that the oil phase with higher density and viscosity slowed down the coalescence process or needed a stronger electric field to complete the coalescence.

The above results presented some insights into the coalescence process and discussed some impact factors; however, more detailed characteristics of the ionic nanodroplets throughout the coalescence process seem to be undeveloped, especially under AC electric field. In this paper, to reveal the unclear microscopic effect mechanism of ions, water molecules, electric field form and strength on the dynamic coalescence and breaking-up processes of the ionic droplets, the molecular dynamic simulation was employed to investigate the coalescence and break-up behaviors of two ionic water droplets under the effect of AC electric field. Furthermore, we systematically discussed the effect of electric field frequency and strength on the processes of droplet deformation, motion and coalescence from molecular perspective. Additionally, the oscillation phenomenon of the coalescence droplet was also found.

## 2. Molecular Simulation Details

**Figure 1:**
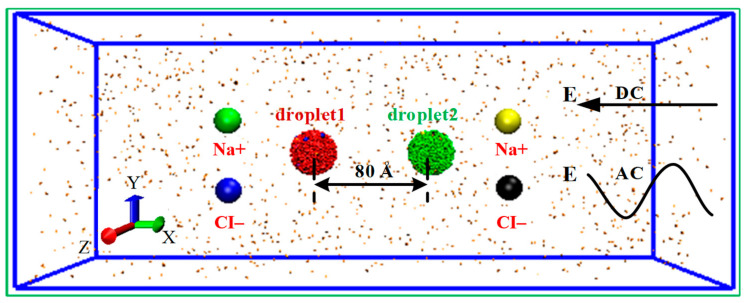
The initial model of the simulated system. DC and AC stands for the direct current (DC) and alternating current (AC) electric field.

To simply the simulation model of separation system, Na+/Cl− ions are applied to represent the general ions in the water droplet. The initial model of the simulated system is shown in Figure 1. Two nanodroplets solved sodium chloride at a concentration of 0.4394 M (each containing 2000 water molecules and 20 Na+ ions, 20 Cl− ions) are placed in the center of the simulation box which was filled with 1200 nitrogen molecules to simulated the other phase. The size of the simulation box is 28 × 70 × 28 nm^3^ and the initial distance between the centroids of the two droplets was 8.0 nm. To demonstrate the interaction and motion relationship of the two droplets more clearly, the droplet at left was numbered as droplet 1 and shown in red, and the right one was numbered as droplet 2 and shown in green. Water molecules are represented by the SPC/E model, which is successfully applied to investigate the wetting behavior of water molecules and other phenomena [30,31,32]. The interactions between water molecules, ions and nitrogen molecules consists of short-range van der Waals interactions and long-range electrostatic interactions. In this study, the classical Lennard-Jones (L-J) potential was adopted to calculate short-range van der Waals interactions, including repulsion and dispersion force, and electrostatic interactions are calculated according to the Coulomb’s law:

(1) $$U_{ij}=\frac{q_{i}q_{j}}{r_{ij}}+4\varepsilon_{ij}\left[{\left(\frac{\sigma_{ij}}{r_{ij}}\right)}^{12}-{\left(\frac{\sigma_{ij}}{r_{ij}}\right)}^{6}\right]$$

where, *q_i_*, *q_j_* is the amount of charge of atoms *i* and *j*, *r_ij_* is the distance between atoms *i* and *j*, and *σ_ij_* and *ε_ij_* represent the scaling parameters and energy parameters of the interaction between atoms *i* and *j*. Table 1 presents all the potential energy parameters and charges for each atom. The potential energy parameter *σ_ij_* and *ε_ij_* between different particles can be calculated according to the Lorentz-Berthelot mixing rule [33] as follows,

(2) $$\varepsilon_{ij}=\sqrt{\varepsilon_{i}\cdot }\varepsilon_{j},\sigma_{ij}=\left(\sigma_{i}+\sigma_{j}\right)/2.0$$

**Figure 2:**
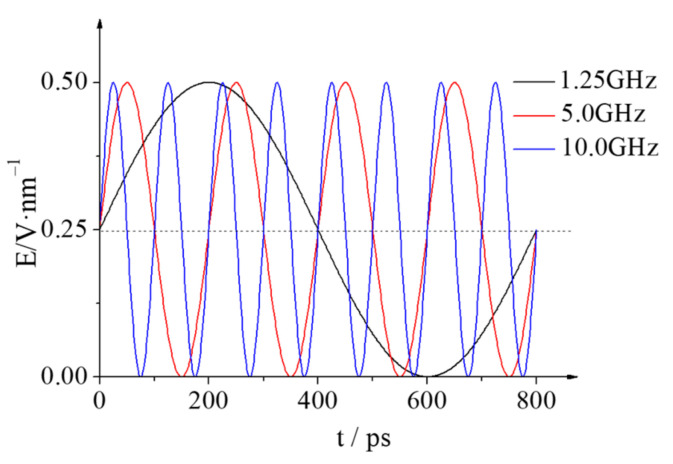
Time evolution of the electric field strength *E* when the electrical frequencies is 1.25, 5.0 and 10.0 GHz (take *E* = 0.50 V/nm for example).

The LAMMPS package [34] was used to simulate the coalescence and separation process of two nanodroplets in the NVT ensemble under the alternative electric field. The cut-off radius of the short-distance L-J potential function was 1.5 nm, and the long-distance electrostatic interactions were solved using the Particle-Particle Particle-Mesh (PPPM) [35] method. The Newtonian equation of motion for each atom was solved using the Velocity-Verlet algorithm [36] with a time step of 1 fs. The Nosé-Hoover thermostat was applied to keep the system at a constant temperature of 298 K. Additionally, periodic boundary conditions were applied in each dimension. When the electric field *E* is applied to the system, each atom is subjected by an external electric field force of *F_ie_* = *Eq_i_* and the direction of the electric field force is determined according to the type of charge *q_i_*. Firstly, the simulation system with the two droplets kept at the initial position was first relaxed for 1 ns without applying an electric field to minimize the energy of the system. Then, several of the AC electric fields of *E* = 0.40~1.50 V/nm with different frequencies are applied to the system to study and analyze the coalescence and separation characteristics of the two nanodroplets, and discuss the effect of the electric field frequency on the critical electric field strength of coalescence and the droplet deformation. Taking the electric field of *E* = 0.50 V/nm as an example, Figure 2 shows the schematic diagram of the electric field E with different frequencies.

## 3. Results and Discussion

### 3.1. Dynamic Coalescence Process of Nanodroplets

Electric field strength has an important impact on the coalescence and separating process of nanodroplets, and furthermore, a critical electric field exists [13,14,15,37]. When the external electric field strength is greater than the critical electric field strength, the droplets no longer merge, or separate with each other after a partial coalescence. In this paper, after the simulation system reaches equilibrium, an AC electric field with a frequency of 1.25, 5.0, and 10.0 GHz—and an electric field strength of 0.4~1.5 V/nm—is applied to analyze the coalescence and separation characteristics of the droplets. During the simulations, the critical electric field strength was gradually determined according to the interpolation method, with a minimum electric field interval of 0.01 V/nm. The corresponding electric field strength at frequency of 1.25, 5.0, and 10.0 GHz is 0.59, 0.71, and 0.77 V/nm, respectively. The critical electric field strength is a little greater than the one under DC electric field when the droplet size is the same [37]. For a macroscopic droplet with radius of 5.0 mm, the critical electric field value is found to be 2000 V/cm in a numerical simulation study [38].

**Figure 3:**
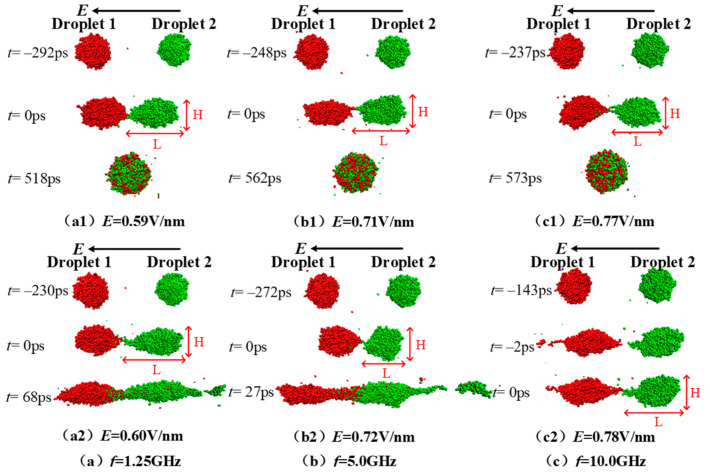
Dynamic coalescence and separation process of the two droplets under electric fields *E* = *E_c_* (shown by subscript 1) and *E* = *E_c_* + 0.01 V/nm (shown by subscript 2) when the electric field frequencies are (**a**) *f* = 1.25 GHz, (**b**) *f* = 5.0 GHz and (**c**) *f* = 10.0 GHz. *L* and *H* represents the length and the height of the droplet.

Figure 3 shows the coalescence process of ionic droplets under the effect of the AC electric field with a frequency of 1.25, 5.0, 10.0 GHz and the electric field strength *E* = *E_c_* and *E* = *E_c_* + 0.01 V/nm. When the AC electric field frequency is 1.25 GHz as seen in Figure 3(a1,a2), droplet 1 and droplet 2 contact with each other and coalescence to 1 droplet after 518 ps under electric field *E* = 0.59 V/nm. While a daughter droplet separates from droplet 2 at the right end after 2 two droplets contacting 68 ps under electric field *E* = 0.60 V/nm, causing partial fracture. According to the physical definition of the critical electric field strength, the critical electric field strength is *E_c_* = 0.59 V/nm at the condition with the AC electric field frequency *f* =1.25 GHz. When the AC field frequency *f* equals 5.0 GHz, the 2 droplets coalescence at 0.71 V/nm and form a big spherical droplet at *t* = 562 ps (as seen in Figure 3(b1)), while when the electric field strength increases by 0.01 V/nm to 0.72 V/nm, a daughter droplet separates from droplet 2 at the right end after the contacting 272 ps. Therefore, the critical electric field strength is *E_c_* = 0.71 V/nm for the case with a frequency of 5.0 GHz. Similarly, it can be seen clearly from the coalescence and separation processes shown in Figure 3(c1,c2) that the corresponding critical electric field strength *E_c_* is 0.77 V/nm for the case with the AC electric field frequency of 10.0 GHz. For a clear presentation, the critical electric field strength *E_c_* and the time required for contacting (*t_0_*) at the condition with three different electric field frequencies, as well as the corresponding information for the case with DC electric field (noted as *f* = 0 GHz) are listed in Table 2. It can be obviously seen from Table 2 that the critical electric field strength *E_c_* increases gradually as the AC field frequency *f* increases, and the time required for contacting (*t_0_*) under the corresponding conditions shows a slight decrease.

### 3.2. Parameter Analysis

**Figure 4:**
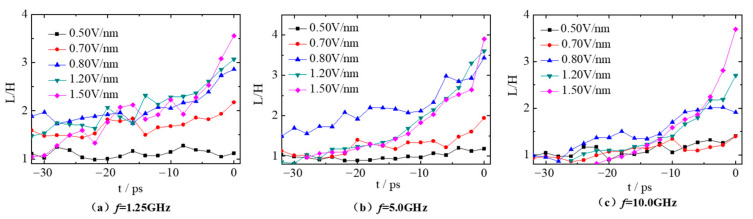
Time evolution of the deformation parameter L/H of droplet 2 under different electric fields with frequency (**a**) *f* = 1.25 GHz, (**b**) *f* = 5.0 GHz and (**c**) *f* = 10.0 GHz.

As can be seen in Figure 3, the droplets are stretched into a spindle in the x direction under the AC electric field. Here, the deformation parameter (L/H) is adopted to characterize the deformation of the droplets. Droplet 1 and droplet 2 are distributed symmetrically in the simulation box at the beginning, and their deformation degree is basically the same. Moreover, the time evolution of the deformation degree of droplet 2 is discussed in this section. Figure 4 gives the deformation parameter L/H of droplet 2 within 32 ps before contacting (−32 ps < *t* < 0 ps) under the electric fields with frequency of 1.25, 5.0, 10.0 GHz and electric field strength of 0.50, 0.70, 0.80, 1.20 and 1.50 V/nm. As can be seen in Figure 4, the corresponding value of L/H fluctuates around 1.0 when the electric field strength *E* is 0.50 V/nm, indicating that droplet 2 is basically spherical throughout the simulation. Therefore, the polarization effect of the electric field on the droplet is very limited at electric field *E* = 0.50 V/nm. However, when the electric field strength increases to 0.70, 0.80, 1.20 and 1.50 V/nm, the deformation of droplet 2 always increases sharply within 6 ps before contacting with the droplet 1 (−6 ps < *t* < 0 ps). This is because the polarization induced by electric fields becomes obvious and the droplets are stretched in the electric field direction.

The deformation of droplets is not only affected by the electric field strength but also by the frequency. From comparative analysis on Figure 4a,b, the deformation curve of droplet 2 fluctuates violently with time when the electric field frequency *f* is 1.25 GHz. There are two main reasons for this. One is that the movement of ions in droplet 2 causes the sharp fluctuation of droplet shape. The other is that droplet 2 has sufficient time to respond to the change of electric field and then the shape deforms accordingly when the electric field frequency f is small. At *t* = −32 ps, the deformation of droplet 2 at the electric field *E* = 0.70, 0.80 and 1.20 V/nm is greater than that at electric fields with frequency of 5.0 GHz, and the deformation degree is 1.59, 1.88 and 1.47, respectively (see Figure 4a). However, when the droplets contact (*t* = 0 ps), the deformation degree is almost the same at electric fields *E* = 0.70, 0.80 and 1.20 V/nm for the 2 cases with frequency of 1.25 GHz and 5.0 GHz. This indicates the droplet 2 deforms less at the case with frequency *f* = 1.25 GHz than that at frequency *f* = 5.0 GHz within the same time period (−32 ps < *t* < 0 ps). As the frequency increases up to 10.0 GHz, the electric field strength changes too fast, droplets are too late to deform accordingly and can only be continuously stretched under the effect of the AC electric field. Thus, the deformation curves of droplet become relatively smoother, especially at *E* = 1.50 V/nm.

**Figure 5:**
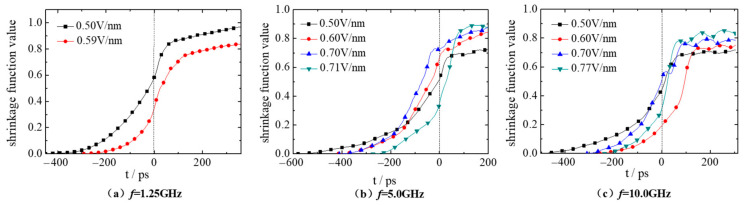
Shrinkage function curve of droplets deformation under AC electric fields with frequency (**a**) *f* = 1.25 GHz, (**b**) *f* = 5.0 GHz and (**c**) *f* = 10.0 GHz.

Furthermore, the centroid trajectory of the droplets during the coalescence process under electric fields can be represented by the shrinkage function, *S*(*t*) [39],

(3) $$S(t)=\Delta L(t)/L_{0}=(L_{0}-L(t))/L_{0}$$

where, *L*_0_ is the initial distance between the centroid of droplet 1 and droplet 2; *L* represents the instantaneous distance between them at time point *t*. Obviously, if the two droplets merge well, the shrinkage function values will tend to 1.0. While the fusion always occurs only in the internal contact region, and atoms in the outer edge cannot move freely under the external electric field and interaction from other atoms. Thus, the shrinkage function values are always less than 1.0. Figure 5 shows the shrinkage function curve during the whole coalescence process of droplet 1 and droplet 2 under electric fields with frequency *f* of 1.25, 5.0 and 10.0 GHz and electric field strength of 0.50, 0.60, 0.70 and *E_c_* V/nm. It should be noted that only two curves with the electric field strength *E* equals 0.50 and 0.59 V/nm are shown in Figure 5a because the critical electric field strength *E_c_* is 0.59 V/nm when *f* is 1.25 GHz. At this case, droplet 1 and droplet 2 coalescence very well under electric field *E* = 0.50 V/nm, and the corresponding shrinkage function value reaches the maximum of 0.97. However, the coalescence effect comes to decrease when the electric field is *E* = *E_c_.* Furthermore, this phenomenon is consistent with the DC electric field [37]. This might be attributed to the following two reasons: the disturbance of the electric field changes to the droplet is obvious when the electric field frequency is low. When the electric field strength is small, the coalescence behavior of droplets mainly relies on the free movement of molecules and ions.

However, when the electric field frequency becomes 5.0, 10.0 GHz (Figure 5b,c), the time required for contacting (*t_0_*) gradually decreases with the increase of electric field strength, and the shrinkage function value of droplets after coalescence is closer to 1. When the *E* = *E_c_*, droplet 1 and droplet 2 perform best in coalescence, with the shrinkage function value of 0.91, 0.83, respectively. Moreover, the time required for contacting (*t_0_*) at 10.0 GHz is smaller than that at 5.0 GHz with the same electric field strength (*E* = 0.50, *E_c_* V/nm), indicating that the increased electric field frequency promotes the droplet movement. In the coalescence process after contacting (i. e., at *t* > 0 ps), the corresponding shrinkage curve value when the electric field frequency *f* is 10.0 GHz is smaller than that at the case with *f* = 5.0 GHz, indicating that the increase of the electric field frequency *f* cannot improve the coalescence effect of the droplets. Table 3 gives the shrinkage function values of the two droplets under electric fields with different electric field strength and frequencies at *t* = 200 ps.

### 3.3. Oscillation Phenomenon after Coalescence

**Figure 6:**
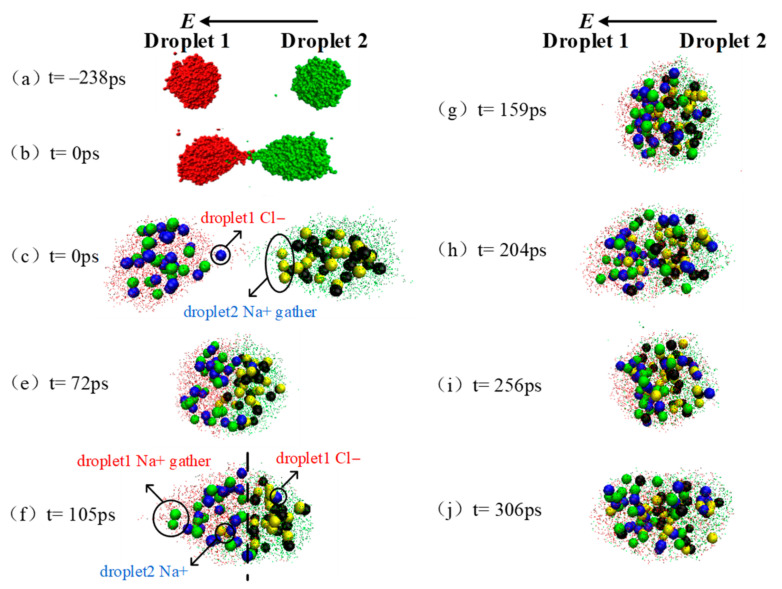
Schematic of droplet coalescence and deformation after contacting at AC electric field (*f* = 10.0 GHz, *E* = 0.77 V/nm).

As discussed above, after the two droplets merged to one big droplet, the shrinkage function curve showed the same fluctuations as the electric field frequency period at *E* = 0.60, 0.70, 0.77 V/nm when the electric field frequency was 10 GHz. Additionally, the largest fluctuation amplitude appears under electric field of 0.77 V/nm. In this section, the dynamic deformation process of the merged droplets are taken as examples to discuss the oscillation phenomenon after convergence. Figure 6 shows the dynamic coalescence process of droplet 1 and droplet 2 under the effect of electric field with *f* = 10.0 GHz, *E* = 0.77 V/nm.

As shown in point (c) in Figure 6, there are ae some ions Cl^−^ in droplet 1 and ions Na^+^ in droplet 2 accumulated at the opposite end under the effect of electric field while the two droplet contacting (*t* = 0 ps). As the two droplets are close to merge, ions Cl^−^ tends to be further mixed to Na^+^ (*t* = 72 ps). At *t* = 105 ps (as seen in Figure 6f), the AC electric field reaches the first peak after coalescence, and the electric field strength reaches the amplitude value. The coalescence droplets are stretched at this moment, resulting in the shrinkage function value being the minimum value of 0.76. The ions that originally start mixing are separated on both sides of the dashed line. At *t* = 159 ps, the AC electric field reaches the first trough after coalescence, the electric field strength reaches the amplitude value in opposite direction. During this process from wave peak to valley, the stretched droplet returns to spherical because of the ions movement under electric field. The corresponding shrinkage function value reaches a big value of 0.84, i. e., the merged droplet looks more spherical.

Subsequently, the merged droplets experience the peak, valley and peak of electric field and the corresponding shrinkage function value reaches to the minimum of 0.79, maximum of 0.85 and minimum of 0.83 at *t* = 204, 256 and 306 ps, respectively. Clearly, the minimum value of shrinkage function become larger as the simulation, indicating a more spherical droplet is formed under this AC electric field. Thus, the merged droplet oscillate along with the AC electric field, and the coalescence effect of droplet 1 and droplet 2 gradually becomes better during the process. 

In conclusion, with the increase of the electric field frequency *f*, the critical electric field strength (*E_c_*) for droplet coalescence increase, and the corresponding time required for droplet contacting (*t*_0_) decreases. The higher the electric field frequency, the less likely the droplet deforms with the periodic change of the AC electric field (*E* = 0.60, 0.70, *E_c_*, 0.80 V/nm). After droplets contact (*t* > 0 ps), the best coalescence of the two droplets occurs under the electric field with frequency of 1.25 GHz and strength of 0.50 V/nm. When the electric field frequency increases to 5.0, 10.0 GHz, the coalescence effect of the droplets shows better with the increase of electric field strength. In addition, at the electric field frequency *f* = 10.0 GHz, the shape of the droplet oscillates obviously after merging, which causes obvious periodic fluctuations of the shrinkage function curve. By investigating the oscillation phenomenon of droplet at *E* = *E_c_*, it was found that the merged droplets are stretched longer at the peak of the AC electric field, the shrinkage function value reach the minimum, while in the valley of AC electric field the large droplets tend to be spherical and the shrinkage function value increase up to the maximum. Finally, the coalescence effect becomes better and a more spherical droplet can form.

## 4. Conclusions

In this paper, the molecular dynamic models of two ionic nanodroplets (NaCl concentration of 0.4394 M) under the AC electric field are established to investigate the coalescence and break-up behaviors of nanodroplet, and to discuss the effect of electric field frequency and strength on the processes of droplet deformation, motion and coalescence. The results show that the time required for the two droplets contacting shows a decrease, and the critical electric field strength *E_c_* increases with the increase of electric field frequency. When the AC electric field frequency is 1.25, 5.0 and 10.0 GHz, the critical electric field strength *E_c_* is 0.59, 0.70 and 0.77 V/nm, respectively. According to the analysis of the shrinkage function, under the electric field *E* = 0.60, 0.70, 0.80, *E_c_* V/nm, the higher the electric field frequency, the less likely the shape of the droplet is to change periodically with the AC electric field. Additionally, a nearly spherical droplet formed under electric field *E* = 0.50 V/nm with *f* = 1.25 GHz. In addition, at the cases with electric field frequency *f* =10.0 GHz, the shape of the droplet oscillates significantly after coalescence, and causes obvious periodic fluctuations in the shrinkage function. The presented simulation results are significant and could provide theoretical basis for the improvement of electrostatic dehydration on the molecular level.

In this paper, the size of the two droplets is the same and nitrogen molecules are applied to represent the other phase (oil). It is better if the real oil phase could be replaced by asphaltene, resin and n-hexane. An excellent outcome may be achieved by considering these effect factors. Therefore, more studies are necessary to reveal the microscopic effect mechanism of droplet size and the real oil structure on the coalescence and break-up behaviors.

## Figures and Tables

**Table 1:** Parameters of the potential function for each atom.

Atom	*σ* (nm)	*ε* (kJ/mol)	*q* (e)
O	0.316	0.6502	−0.8476
H	0	0	0.4238
Na	0.258	0.6173	+1.0000
Cl	0.440	1.0512	−1.0000
N	0.331	0.7908	+0.0000

**Table 2:** Electric field frequency *f* dependents of critical electric field strength (*E*_c_) and time required for contacting (*t*_0_).

Electric Field Frequency *f*/GHz	Critical Electric Field Strength *E_c_*/V·nm^−1^	Time Required for Contacing *t*_0_/ps
0 [33]	0.52	130
1.25	0.59	292
5.0	0.71	248
10.0	0.77	237

**Table 3:** The shrinkage function values of the two droplets under electric fields with different strengths and frequencies at *t* = 200 ps.

Electric Field Strength *E*_c_ V·nm^−1^	Electric Field Frequency *f* = 5.0 GHz	Electric Field Frequency *f* = 10.0 GHz
0.50	0.73	0.71
0.60	0.85	0.72
0.70	0.88	0.79
0.71 (*f* = 5.0 GHz) 0.77 (*f* = 10.0 GHz)	0.88	0.81

## Data Availability

Not applicable.
